# 1,3,3-Tribenzyl­indolin-2-one

**DOI:** 10.1107/S1600536811042425

**Published:** 2011-10-22

**Authors:** Yufang Liu, Bo Liu, Zhenming Dong, Shuo Jin

**Affiliations:** a School of Chemistry and Chemical Engineering, Shanxi University, Taiyuan 030006, Shanxi Province, People’s Republic of China; b School of Science, Beijing Jiaotong University, Beijing 100044, People’s Republic of China

## Abstract

In the title compound, C_29_H_25_NO, the dihedral angles between the indolin-2-one ring system and the three benzene rings are 62.78 (9), 31.69 (9) and 80.94 (9)°.

## Related literature

For general background to the use of indoline-2-one compounds as precursors for the synthesis of anti­tumor agents, see: Wang *et al.* (2011 ▶). For a related structure, see: Katritzky *et al.* (1997 ▶).
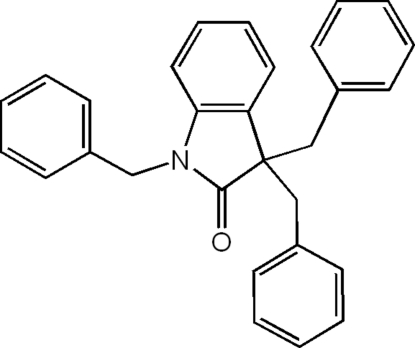


## Experimental

### 

#### Crystal data

C_29_H_25_NO
*M* *_r_* = 403.50
Monoclinic, 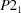

*a* = 8.3387 (9) Å
*b* = 9.6266 (10) Å
*c* = 13.9398 (14) Å
β = 99.442 (2)°
*V* = 1103.8 (2) Å^3^
*Z* = 2
Mo *K*α radiation
μ = 0.07 mm^−1^
*T* = 296 K
0.30 × 0.20 × 0.20 mm

#### Data collection

Bruker SMART APEX CCD area-detector diffractometer
Absorption correction: multi-scan (*SADABS*; Sheldrick, 1996 ▶) *T*_min_ = 0.979, *T*_max_ = 0.986
4797 measured reflections
2073 independent reflections
1731 reflections with *I* > 2σ(*I*)
*R*_int_ = 0.028

#### Refinement

*R*[*F* ^2^ > 2σ(*F* ^2^)] = 0.033
*wR*(*F* ^2^) = 0.076
*S* = 1.05
2073 reflections
281 parameters
1 restraint
H-atom parameters constrained
Δρ_max_ = 0.10 e Å^−3^
Δρ_min_ = −0.11 e Å^−3^

### 

Data collection: *SMART* (Siemens, 1996 ▶); cell refinement: *SAINT* (Siemens, 1996 ▶); data reduction: *SAINT*; program(s) used to solve structure: *SHELXS97* (Sheldrick, 2008 ▶); program(s) used to refine structure: *SHELXL97* (Sheldrick, 2008 ▶); molecular graphics: *ORTEP-3 for Windows* (Farrugia, 1997 ▶); software used to prepare material for publication: *SHELXL97*.

## Supplementary Material

Crystal structure: contains datablock(s) global, I. DOI: 10.1107/S1600536811042425/hg5107sup1.cif

Structure factors: contains datablock(s) I. DOI: 10.1107/S1600536811042425/hg5107Isup2.hkl

Supplementary material file. DOI: 10.1107/S1600536811042425/hg5107Isup3.cml

Additional supplementary materials:  crystallographic information; 3D view; checkCIF report

## Figures and Tables

**Fig. 1.**
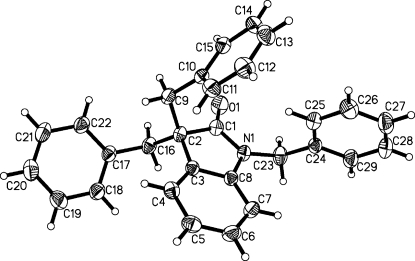
The molecular structure of the title compound with the atom numbering scheme. Displacement ellipsoids are drawn at the 30% probability level.

**Fig. 2.**
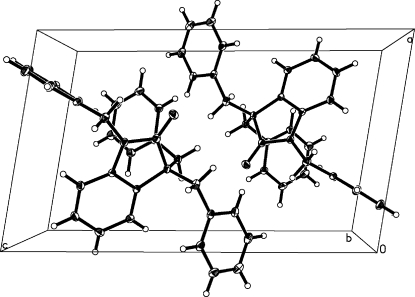
The molecular packing of (I) viewed along the b axis.

## supplementary crystallographic information

### Comment

Indoline-2-one compounds have been widely explored as precursors for the synthesis of antitumor agent (Wang *et al.*, 2011). In the course of exploring new antitumor medicine, we obtained a intermediate compound C_29_H_25_NO (I), the synthesis and structure of which are reported here.

The title compound contain four ring planes, three benzene rings and one indoline-2-one ring. The interplanar dihedral angle between the indolin-2-one ring plane and the three benzene ring planes are 62.78 (9)°, 31.69 (9)° and 80.94 (9)\ respectively.

The molecules of (I) crystallize in the space group *P*2_1_ which is different from that of 3-(1,2-diphenylethylidene)indolin-2-one (*P*-1) (Katritzky *et al.* 1997).

### Experimental

Indolin-2-one (0.50 g, 3.76 mmol) was dissolved in THF (20 mL) and KOH (0.80 g, 14.3 mmol) was slowly added. After heating the stirred mixture at reflux temperature for 30 min, a solution of 1-(chloromethyl)benzene (2.00 g, 15.9 mmol) in THF was slowly added and the refluxing continued for 2 h. The mixture was then cooled to 333 K and poured into water (200 mL) and was extracted with chloroform and dried over Na_2_SO_4_. After removing the solvent, the crude product was purified by column chromatography on silica gel, affording the title compound (yield: 0.23 g, 15%). The compound was then dissolved in THF, and colorless crystals were formed on slow evaporation at room temperature over one week.

### Refinement

All H atoms were placed in geometrically calculated positions and refined using a riding model with C—H = 0.93 Å and with *U*_iso_(H) = 1.2*U*_eq_(C).

### Crystal data

**Table:**

C_29_H_25_NO	*F*(000) = 428
*M**_r_* = 403.50	*D*_x_ = 1.214 Mg m^−^^3^
Monoclinic, *P*2_1_	Mo *K*α radiation, λ = 0.71073 Å
Hall symbol: P 2yb	Cell parameters from 2492 reflections
*a* = 8.3387 (9) Å	θ = 2.9–26.3°
*b* = 9.6266 (10) Å	µ = 0.07 mm^−^^1^
*c* = 13.9398 (14) Å	*T* = 296 K
β = 99.442 (2)°	Block, yellow
*V* = 1103.8 (2) Å^3^	0.30 × 0.20 × 0.20 mm
*Z* = 2	

### Data collection

**Table:**

Bruker SMART APEX CCD area-detector diffractometer	2073 independent reflections
Radiation source: fine-focus sealed tube	1731 reflections with *I* > 2σ(*I*)
graphite	*R*_int_ = 0.028
phi and ω scans	θ_max_ = 25.1°, θ_min_ = 1.5°
Absorption correction: multi-scan (*SADABS*; Sheldrick, 1996)	*h* = −9→7
*T*_min_ = 0.979, *T*_max_ = 0.986	*k* = −11→11
4797 measured reflections	*l* = −16→16

### Refinement

**Table:**

Refinement on *F*^2^	Secondary atom site location: difference Fourier map
Least-squares matrix: full	Hydrogen site location: inferred from neighbouring sites
*R*[*F*^2^ > 2σ(*F*^2^)] = 0.033	H-atom parameters constrained
*wR*(*F*^2^) = 0.076	*w* = 1/[σ^2^(*F*_o_^2^) + (0.0404*P*)^2^] where *P* = (*F*_o_^2^ + 2*F*_c_^2^)/3
*S* = 1.05	(Δ/σ)_max_ < 0.001
2073 reflections	Δρ_max_ = 0.10 e Å^−^^3^
281 parameters	Δρ_min_ = −0.11 e Å^−^^3^
1 restraint	Extinction correction: *SHELXL97* (Sheldrick, 2008), Fc^*^=kFc[1+0.001xFc^2^λ^3^/sin(2θ)]^-1/4^
Primary atom site location: structure-invariant direct methods	Extinction coefficient: 0.085 (5)

### Special details

**Table:**

Geometry. All esds (except the esd in the dihedral angle between two l.s. planes) are estimated using the full covariance matrix. The cell esds are taken into account individually in the estimation of esds in distances, angles and torsion angles; correlations between esds in cell parameters are only used when they are defined by crystal symmetry. An approximate (isotropic) treatment of cell esds is used for estimating esds involving l.s. planes.
Refinement. Refinement of F^2^ against ALL reflections. The weighted R-factor wR and goodness of fit S are based on F^2^, conventional R-factors R are based on F, with F set to zero for negative F^2^. The threshold expression of F^2^ > 2sigma(F^2^) is used only for calculating R-factors(gt) etc. and is not relevant to the choice of reflections for refinement. R-factors based on F^2^ are statistically about twice as large as those based on F, and R- factors based on ALL data will be even larger.

### Fractional atomic coordinates and isotropic or equivalent isotropic displacement parameters (Å^2^)

**Table:**

	*x*	*y*	*z*	*U*_iso_*/*U*_eq_	
O1	0.3706 (2)	−0.1015 (2)	0.38221 (12)	0.0693 (5)	
N1	0.5070 (2)	−0.1315 (2)	0.25382 (12)	0.0454 (5)	
C24	0.3103 (3)	−0.1520 (2)	0.09974 (16)	0.0446 (6)	
C8	0.6583 (2)	−0.0925 (2)	0.23014 (14)	0.0415 (5)	
C3	0.7426 (3)	−0.0128 (2)	0.30485 (14)	0.0393 (5)	
C16	0.7158 (3)	−0.0745 (3)	0.48023 (15)	0.0519 (6)	
H16A	0.6343	−0.0772	0.5225	0.062*	
H16B	0.7383	−0.1698	0.4640	0.062*	
C10	0.4948 (3)	0.2266 (2)	0.32102 (16)	0.0464 (6)	
C15	0.3277 (3)	0.2368 (3)	0.31634 (18)	0.0558 (7)	
H15	0.2784	0.1947	0.3640	0.067*	
C4	0.8954 (3)	0.0370 (3)	0.29641 (17)	0.0503 (6)	
H4	0.9531	0.0917	0.3452	0.060*	
C2	0.6413 (3)	0.0012 (2)	0.38491 (15)	0.0437 (6)	
C1	0.4884 (3)	−0.0807 (3)	0.34282 (16)	0.0468 (6)	
C25	0.3007 (3)	−0.0100 (3)	0.08741 (17)	0.0524 (6)	
H25	0.3496	0.0480	0.1371	0.063*	
C17	0.8693 (3)	−0.0135 (2)	0.53710 (15)	0.0488 (6)	
C23	0.3890 (3)	−0.2184 (3)	0.19369 (17)	0.0574 (7)	
H23A	0.3046	−0.2437	0.2308	0.069*	
H23B	0.4424	−0.3033	0.1787	0.069*	
C18	1.0215 (3)	−0.0589 (3)	0.52212 (17)	0.0612 (7)	
H18	1.0286	−0.1267	0.4755	0.073*	
C9	0.5968 (3)	0.1523 (3)	0.40544 (16)	0.0504 (6)	
H9A	0.5382	0.1527	0.4601	0.060*	
H9B	0.6965	0.2043	0.4247	0.060*	
C22	0.8643 (3)	0.0867 (3)	0.60759 (16)	0.0566 (7)	
H22	0.7641	0.1195	0.6188	0.068*	
C6	0.8748 (3)	−0.0747 (3)	0.14105 (18)	0.0610 (7)	
H6	0.9211	−0.0958	0.0865	0.073*	
C29	0.2389 (3)	−0.2353 (3)	0.02380 (18)	0.0629 (7)	
H29	0.2455	−0.3314	0.0302	0.075*	
C26	0.2199 (3)	0.0472 (3)	0.0027 (2)	0.0668 (8)	
H26	0.2133	0.1432	−0.0042	0.080*	
C7	0.7211 (3)	−0.1231 (3)	0.14706 (16)	0.0547 (6)	
H7	0.6619	−0.1745	0.0969	0.066*	
C11	0.5633 (3)	0.2891 (3)	0.24806 (17)	0.0578 (7)	
H11	0.6752	0.2844	0.2500	0.069*	
C21	1.0043 (4)	0.1389 (3)	0.66143 (19)	0.0682 (8)	
H21	0.9976	0.2061	0.7085	0.082*	
C12	0.4683 (3)	0.3586 (3)	0.1721 (2)	0.0697 (8)	
H12	0.5161	0.3982	0.1229	0.084*	
C20	1.1531 (4)	0.0928 (3)	0.6464 (2)	0.0698 (8)	
H20	1.2474	0.1271	0.6838	0.084*	
C5	0.9610 (3)	0.0040 (3)	0.21408 (18)	0.0580 (7)	
H5	1.0644	0.0355	0.2083	0.070*	
C28	0.1581 (3)	−0.1783 (4)	−0.0613 (2)	0.0754 (9)	
H28	0.1100	−0.2359	−0.1114	0.090*	
C14	0.2335 (3)	0.3088 (3)	0.2416 (2)	0.0639 (7)	
H14	0.1220	0.3166	0.2403	0.077*	
C13	0.3036 (3)	0.3686 (3)	0.1697 (2)	0.0684 (8)	
H13	0.2396	0.4160	0.1192	0.082*	
C19	1.1621 (3)	−0.0052 (3)	0.5751 (2)	0.0710 (8)	
H19	1.2629	−0.0350	0.5628	0.085*	
C27	0.1485 (3)	−0.0374 (4)	−0.0721 (2)	0.0730 (9)	
H27	0.0942	0.0012	−0.1296	0.088*	

### Atomic displacement parameters (Å^2^)

**Table:**

	*U*^11^	*U*^22^	*U*^33^	*U*^12^	*U*^13^	*U*^23^
O1	0.0624 (10)	0.0862 (14)	0.0635 (10)	−0.0224 (10)	0.0228 (9)	−0.0056 (11)
N1	0.0469 (11)	0.0475 (12)	0.0406 (10)	−0.0077 (9)	0.0037 (8)	−0.0046 (9)
C24	0.0411 (12)	0.0487 (15)	0.0428 (13)	−0.0069 (11)	0.0039 (10)	−0.0058 (11)
C8	0.0445 (12)	0.0402 (13)	0.0387 (11)	0.0010 (11)	0.0037 (10)	0.0007 (11)
C3	0.0425 (12)	0.0363 (12)	0.0375 (11)	0.0003 (10)	0.0016 (9)	0.0013 (10)
C16	0.0670 (15)	0.0468 (14)	0.0406 (12)	−0.0041 (13)	0.0048 (11)	0.0017 (12)
C10	0.0506 (14)	0.0403 (13)	0.0462 (13)	0.0024 (11)	0.0014 (11)	−0.0099 (11)
C15	0.0539 (15)	0.0581 (16)	0.0539 (14)	−0.0019 (13)	0.0041 (12)	−0.0131 (14)
C4	0.0485 (14)	0.0491 (15)	0.0516 (14)	−0.0032 (11)	0.0027 (11)	0.0018 (12)
C2	0.0498 (13)	0.0458 (14)	0.0346 (11)	−0.0052 (11)	0.0048 (10)	−0.0015 (11)
C1	0.0499 (13)	0.0468 (14)	0.0434 (12)	−0.0058 (12)	0.0067 (11)	0.0027 (12)
C25	0.0567 (15)	0.0540 (16)	0.0462 (14)	−0.0064 (12)	0.0073 (11)	−0.0014 (13)
C17	0.0633 (15)	0.0434 (14)	0.0367 (11)	0.0057 (12)	−0.0003 (11)	0.0033 (11)
C23	0.0633 (16)	0.0510 (16)	0.0540 (14)	−0.0171 (13)	−0.0019 (12)	−0.0020 (13)
C18	0.0718 (17)	0.0545 (16)	0.0521 (15)	0.0139 (14)	−0.0049 (13)	−0.0046 (13)
C9	0.0571 (14)	0.0504 (15)	0.0425 (13)	0.0005 (12)	0.0047 (11)	−0.0086 (12)
C22	0.0729 (17)	0.0547 (16)	0.0402 (13)	0.0072 (13)	0.0035 (12)	−0.0010 (13)
C6	0.0639 (16)	0.0716 (18)	0.0511 (13)	0.0114 (15)	0.0203 (13)	−0.0025 (14)
C29	0.0678 (17)	0.0570 (17)	0.0587 (16)	−0.0054 (14)	−0.0047 (13)	−0.0108 (14)
C26	0.0690 (17)	0.0654 (18)	0.0652 (18)	−0.0027 (15)	0.0081 (14)	0.0138 (15)
C7	0.0608 (15)	0.0583 (16)	0.0446 (12)	0.0040 (13)	0.0075 (11)	−0.0074 (13)
C11	0.0532 (15)	0.0559 (16)	0.0630 (16)	−0.0005 (13)	0.0061 (13)	0.0048 (14)
C21	0.095 (2)	0.0543 (16)	0.0498 (15)	−0.0009 (16)	−0.0057 (15)	−0.0088 (14)
C12	0.0756 (19)	0.0597 (17)	0.0717 (17)	0.0039 (15)	0.0063 (15)	0.0158 (15)
C20	0.0749 (19)	0.0567 (18)	0.0686 (18)	−0.0057 (15)	−0.0152 (15)	0.0032 (16)
C5	0.0512 (14)	0.0616 (17)	0.0641 (16)	0.0033 (13)	0.0176 (13)	0.0075 (14)
C28	0.077 (2)	0.089 (2)	0.0528 (17)	−0.0057 (18)	−0.0112 (14)	−0.0182 (17)
C14	0.0502 (15)	0.0652 (17)	0.0713 (17)	0.0067 (14)	−0.0049 (13)	−0.0105 (15)
C13	0.0723 (19)	0.0517 (16)	0.0732 (18)	0.0061 (15)	−0.0120 (15)	0.0052 (16)
C19	0.0628 (17)	0.0706 (19)	0.0736 (17)	0.0133 (15)	−0.0062 (14)	0.0036 (17)
C27	0.0704 (19)	0.097 (3)	0.0476 (15)	0.0017 (17)	−0.0012 (13)	0.0128 (17)

### Geometric parameters (Å, °)

**Table:**

O1—C1	1.218 (3)	C18—C19	1.380 (4)
N1—C1	1.366 (3)	C18—H18	0.9300
N1—C8	1.407 (3)	C9—H9A	0.9700
N1—C23	1.449 (3)	C9—H9B	0.9700
C24—C25	1.379 (3)	C22—C21	1.375 (3)
C24—C29	1.383 (3)	C22—H22	0.9300
C24—C23	1.507 (3)	C6—C5	1.374 (4)
C8—C7	1.379 (3)	C6—C7	1.380 (3)
C8—C3	1.388 (3)	C6—H6	0.9300
C3—C4	1.384 (3)	C29—C28	1.379 (4)
C3—C2	1.512 (3)	C29—H29	0.9300
C16—C17	1.510 (3)	C26—C27	1.379 (4)
C16—C2	1.553 (3)	C26—H26	0.9300
C16—H16A	0.9700	C7—H7	0.9300
C16—H16B	0.9700	C11—C12	1.386 (3)
C10—C11	1.383 (3)	C11—H11	0.9300
C10—C15	1.388 (3)	C21—C20	1.366 (4)
C10—C9	1.514 (3)	C21—H21	0.9300
C15—C14	1.384 (3)	C12—C13	1.372 (4)
C15—H15	0.9300	C12—H12	0.9300
C4—C5	1.387 (3)	C20—C19	1.381 (4)
C4—H4	0.9300	C20—H20	0.9300
C2—C1	1.532 (3)	C5—H5	0.9300
C2—C9	1.539 (3)	C28—C27	1.365 (4)
C25—C26	1.375 (3)	C28—H28	0.9300
C25—H25	0.9300	C14—C13	1.367 (4)
C17—C22	1.383 (3)	C14—H14	0.9300
C17—C18	1.389 (3)	C13—H13	0.9300
C23—H23A	0.9700	C19—H19	0.9300
C23—H23B	0.9700	C27—H27	0.9300
			
C1—N1—C8	110.93 (17)	C10—C9—C2	115.07 (18)
C1—N1—C23	124.39 (19)	C10—C9—H9A	108.5
C8—N1—C23	124.65 (19)	C2—C9—H9A	108.5
C25—C24—C29	118.1 (2)	C10—C9—H9B	108.5
C25—C24—C23	122.5 (2)	C2—C9—H9B	108.5
C29—C24—C23	119.4 (2)	H9A—C9—H9B	107.5
C7—C8—C3	122.2 (2)	C21—C22—C17	121.4 (3)
C7—C8—N1	128.2 (2)	C21—C22—H22	119.3
C3—C8—N1	109.58 (17)	C17—C22—H22	119.3
C4—C3—C8	119.18 (19)	C5—C6—C7	121.3 (2)
C4—C3—C2	131.7 (2)	C5—C6—H6	119.3
C8—C3—C2	109.10 (17)	C7—C6—H6	119.3
C17—C16—C2	116.90 (19)	C28—C29—C24	121.1 (3)
C17—C16—H16A	108.1	C28—C29—H29	119.5
C2—C16—H16A	108.1	C24—C29—H29	119.5
C17—C16—H16B	108.1	C25—C26—C27	120.2 (3)
C2—C16—H16B	108.1	C25—C26—H26	119.9
H16A—C16—H16B	107.3	C27—C26—H26	119.9
C11—C10—C15	117.9 (2)	C8—C7—C6	117.6 (2)
C11—C10—C9	122.0 (2)	C8—C7—H7	121.2
C15—C10—C9	120.1 (2)	C6—C7—H7	121.2
C14—C15—C10	120.8 (3)	C10—C11—C12	121.2 (2)
C14—C15—H15	119.6	C10—C11—H11	119.4
C10—C15—H15	119.6	C12—C11—H11	119.4
C3—C4—C5	119.0 (2)	C20—C21—C22	120.6 (3)
C3—C4—H4	120.5	C20—C21—H21	119.7
C5—C4—H4	120.5	C22—C21—H21	119.7
C3—C2—C1	101.66 (17)	C13—C12—C11	119.8 (3)
C3—C2—C9	113.73 (18)	C13—C12—H12	120.1
C1—C2—C9	110.30 (19)	C11—C12—H12	120.1
C3—C2—C16	113.32 (18)	C21—C20—C19	119.4 (3)
C1—C2—C16	106.07 (17)	C21—C20—H20	120.3
C9—C2—C16	111.05 (18)	C19—C20—H20	120.3
O1—C1—N1	124.8 (2)	C6—C5—C4	120.7 (2)
O1—C1—C2	126.4 (2)	C6—C5—H5	119.7
N1—C1—C2	108.73 (19)	C4—C5—H5	119.7
C26—C25—C24	120.9 (2)	C27—C28—C29	120.2 (3)
C26—C25—H25	119.5	C27—C28—H28	119.9
C24—C25—H25	119.5	C29—C28—H28	119.9
C22—C17—C18	117.4 (2)	C13—C14—C15	120.3 (2)
C22—C17—C16	121.5 (2)	C13—C14—H14	119.8
C18—C17—C16	121.0 (2)	C15—C14—H14	119.8
N1—C23—C24	114.57 (19)	C14—C13—C12	119.9 (3)
N1—C23—H23A	108.6	C14—C13—H13	120.0
C24—C23—H23A	108.6	C12—C13—H13	120.0
N1—C23—H23B	108.6	C20—C19—C18	119.9 (3)
C24—C23—H23B	108.6	C20—C19—H19	120.0
H23A—C23—H23B	107.6	C18—C19—H19	120.0
C19—C18—C17	121.3 (3)	C28—C27—C26	119.5 (3)
C19—C18—H18	119.4	C28—C27—H27	120.2
C17—C18—H18	119.4	C26—C27—H27	120.2
